# A Case of Possible Neurosarcoidosis Presenting as Intractable Headache and Panhypopituitarism

**DOI:** 10.1155/2013/816236

**Published:** 2013-08-07

**Authors:** Jin Kyung Hwang, Joo Hee Cho, So Young Park, Jung Il Son, Uk Jo, Sang Ouk Chin, Yun Jung Lee, Moon Chan Choi, Sang Youl Rhee, Eui Jong Kim, Suk Chon

**Affiliations:** ^1^Department of Endocrinology and Metabolism, Kyung Hee University School of Medicine, No. 1 Hoegi-dong, Dongdaemun-gu, Seoul 130-702, Republic of Korea; ^2^Department of Radiology, Kyung Hee University School of Medicine, No. 1 Hoegi-dong, Dongdaemun-gu, Seoul 130-702, Republic of Korea

## Abstract

Sarcoidosis is a chronic multisystemic inflammatory disease of unknown etiology, which is characterized by noncaseating granulomatous inflammation of the involved organs. It is known that neurosarcoidosis involving the nervous system occurs in about 5% of patients with sarcoidosis. However, neurosarcoidosis without systemic involvement is extremely rare. We present a case of suspicious neurosarcoidosis affecting the pituitary gland, which was manifested as chronic uncontrolled headache, panhypopituitarism, central diabetes insipidus, and hypercalcemia. Though the biopsy at the pituitary lesion was not performed due to the high risk of surgical complication, treatment was needed urgently and we started steroid therapy. After steroid therapy, we observed the immediate symptom relief with improved hypercalcemia. According to the follow-up examination, no recurrent symptom was seen, and resolution of the pituitary lesion with improving panhypopituitarism was noted.

## 1. Introduction

Sarcoidosis is a noncaseating granulomatous disease that can affect any system of the body [1]. Neurosarcoidosis involves the nervous system and occurs in less than 5% of patients with systemic sarcoidosis. Isolated neurosarcoidosis without systemic involvement is extremely rare [2].

We describe a case of possible neurosarcoidosis of the pituitary gland without any other systemic involvement. The patient presented with panhypopituitarism, central diabetes insipidus, and hypercalcemia. Sellar magnetic resonance imaging (MRI) showed an infiltrating mass of the pituitary gland with invasion of stalk and cavernous sinus. Steroid therapy resulted in the resolution of the pituitary lesion on sellar MRI and all symptoms.

## 2. Case Presentation

**Figure 1:**
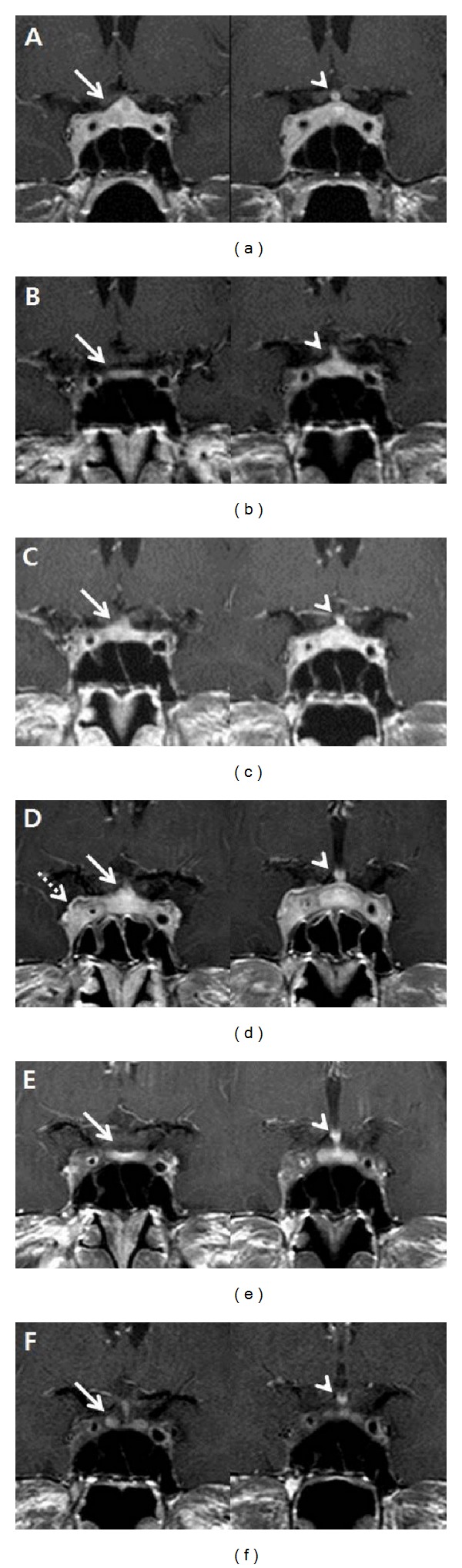
Serial follow-up sellar magnetic resonance image (MRI, contrast enhanced T1-weighted image): (a) initial contrast enhanced image showed suspicious mass of 8 mm in size at right pituitary (arrow, nonenhanced portion) and left sided stalk deviation with thickening (arrowhead). (b) Follow-up MRI obtained after 2 years of intermittent empirical steroid therapy for intractable headache showed reduction in the size of pituitary mass (arrow) and improvement of stalk deviation (arrowhead). (c) Follow-up MRI obtained 4 years after initial examination before gamma knife radiosurgery showed regrowth of pituitary mass with stalk deviation (arrow). (d) Follow-up MRI 1 year after gamma knife radiosurgery before high-dose steroid therapy showed remarkable enlargement of the pituitary mass with thickening and deviation of stalk (arrow) and invasion to cavernous sinus (dotted arrow). Significant stenosis of the right cavernous sinus internal carotid artery was present (arrowhead). (e) MRI obtained at 3 months after high-dose steroid therapy showed marked decline in the size of mass at pituitary gland and cavernous sinus (arrow). There was still visible luminal narrowing of cavernous internal carotid artery (arrowhead). There was also less soft tissue surrounding the right internal carotid artery. (f) MRI obtained at 48 months after high-dose steroid therapy showed reduction in the size of the pituitary and cavernous sinus mass (arrow). Thickening of the pituitary stalk was also decreased (arrowhead), and the soft tissue surrounding the right internal carotid artery was improved.

A 39-year-old man presented to our clinic with nausea and headache. He reported that these symptoms had waxed and waned for five years and were aggravated for three weeks before admission. His medical history revealed that he had been diagnosed with a pituitary microadenoma at the age of thirty four with symptoms of polyuria, polydipsia, and severe headache. Initial sellar MRI showed a pituitary microadenoma with stalk deviation and thickening (Figure 1(a)). An anterior pituitary function test revealed minimal elevation of prolactin (20.2 ng/mL) with no other significant abnormalities. He began to take lisuride hydrogen maleate, which decreased his serum prolactin level. Although intranasal vasopressin therapy significantly relieved the polyuria and polydipsia, his headache did not subside, and, thus, intermittent low-dose steroid therapy was performed based on clinical evidence suggesting that corticosteroids can treat migraine and cluster headache [3]. Interestingly, his headache responded effectively to steroid therapy, and the size of the pituitary mass was decreased with improvement of stalk deviation as compared to the initial sellar MRI (Figure 1(b)).

After discharge, the patient had a symptom-free period of three years with intermittent steroid therapy. However, nine months before the current admission, his headache recurred with severe olfactory-induced emesis. These symptoms did not respond to steroid therapy as previously. Due to the enlargement of the pituitary tumor on the follow-up sellar MRI, gamma knife radiosurgery was performed (Figure 1(c)), but his symptoms aggravated. 

When readmitted because of progressive worsening of the symptoms, he was presenting with general weakness and weight loss of 13 kg during the previous six months (from 66 kg to 53 kg) without sign of dehydration, skin rash, or pigmentation. Galactorrhea was not observed. The testes retained a normal size and consistency, but beard was decreased. His visual field was intact, and there was no focal neurologic abnormality.

The patient's serum creatinine level was 1.3 mg/dL, and serum calcium was 12.1 mg/dL. His 24-hour urinary calcium was above normal (484.77 mg/day). Parathyroid hormone (PTH) was lower than normal (5.7 pg/mL), while PTH-related peptide (PTHrP) was normal (1.1 pmol/L). The level of 1,25-dihydroxyvitamin D was lower than normal (3.3 pg/mL), while the level of 25-hydroxyvitamin D was normal (11.5 ng/mL). A thyroid function test revealed normal levels of free T4, T3 (free T4 1.64 *μ*g/dL, T3 204 ng/dL) and low level of TSH (0.06 *μ*U/mL). Thyrotropin binding inhibitory immunoglobulins (TBII) was 13.9%. Combined pituitary stimulation tests were consistent with panhypopituitarism. The results of a tuberculin skin test and interferon-gamma release assays for latent tuberculosis infection were negative. The level of angiotensin converting enzyme (ACE) was normal (17 U/L). There was no evidence of infectious disease.

**Figure 2:**
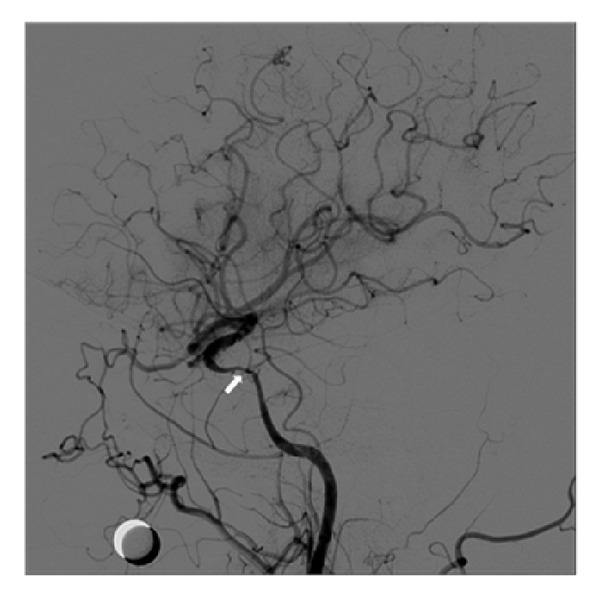
Digital subtraction cerebral angiography: moderate stenosis at right cavernous internal carotid artery with ulceration.

His follow-up brain MRI indicated that the lesion initially regarded as a pituitary microadenoma had become larger (13 mm) with contrast enhanced T1-weighted images. In addition, the mass was extended into the right cavernous sinus and caused narrowing of the internal carotid artery, as confirmed by the digital subtraction cerebral angiography (Figure 2). Thickening of the pituitary stalk with deviation to the left also reappeared (Figure 1(d)). After a thorough radiologic review of previous MRI findings and the clinical and laboratory data, we determined that the pituitary lesion was the result of an inflammatory disease, such as neurosarcoidosis or lymphocytic hypophysitis.

Surgical biopsy for diagnostic confirmation was postponed due to the small size of the mass and stenosis of the right cavernous internal carotid artery, both of which could cause serious surgical complications. Instead, high-dose steroid therapy was planned to resolve the impending obstruction of the internal carotid arteries and severe headache with the presumptive diagnosis of neurosarcoidosis.

Saline hydration lowered the patient's serum creatinine level to 1.0 mg/dL, but hypercalcemia was not easily corrected even after several days of adequate hydration. Oral administration of 300 mg hydrocortisone rapidly corrected the hypercalcemia to 8.0–9.0 mg/dL, and the symptoms of headache and olfactory-induced emesis were also relieved. Given the characteristic findings of the sellar MRI and the resolution of the patient's hypercalcemia after steroid therapy, panhypopituitarism, and central diabetes insipidus, neurosarcoidosis of the pituitary gland was strongly suggested. 

After three months of prednisolone therapy with dose tapering, an apparent reduction in size of the infiltrating pituitary lesion was observed on sellar MRI (Figure 1(e)). After nine months, the patient's combined pituitary stimulation tests showed improvement of the anterior pituitary function with no need for hormone replacement therapy. The patient is currently taking desmopressin and oral calcium carbonate. Sellar MRI at forty-eight months revealed no evidence of recurrence (Figure 1(f)). 

## 3. Discussion 

Sarcoidosis is a chronic inflammatory disease of unknown etiology that is characterized by noncaseating granulomatous inflammation and involvement of almost entire system of the body [1]. In Republic of Korea, about 1.3% of sarcoidosis cases involve the nervous system [4].

The diagnosis of neurosarcoidosis should satisfy three components. First, characteristic clinical and radiologic findings should be evident. Second, other diseases, including vasculitis, infection, and neoplasm, should be excluded. Finally, noncaseating granulomatous inflammation should be histologically confirmed [5]. Hypercalcemia is present in about 13 to 20% of cases. Sarcoidal granulomas produce ACE which is elevated in about 60% of patients with sarcoidosis. However, the value of serum ACE levels in the diagnosis of sarcoidosis remains controversial due to its inadequate sensitivity and specificity [6].

Intracranial neurosarcoidosis often affects the leptomeninges and commonly presents as nodular and/or diffuse infiltrative growing lesions [7]. Less commonly, neurosarcoidosis may appear as an intracranial mass lesion on MRI. Large sarcoid masses within the brain parenchyma are isointense on T1-weighted images and hyperintense on T2-weighted images and also present with nodular or annular enhancement [8]. The sellar MRI findings of our patient showed a focal bulging contour with right cavernous sinus invasion, delayed enhancement of the pituitary gland, and thickening of the pituitary stalk (Figures 1(a) and 1(d)). It is reasonable to assume that the mass-like lesion that had been initially regarded as a pituitary microadenoma was actually an intracranial sarcoid granuloma. Although lymphocytic hypophysitis can have similar symptoms and radiologic findings, it usually regresses spontaneously. The features of the MRI indicative of lymphocytic hypophysitis include loss of the hyperintense “bright spot” signal of the posterior pituitary on T1-weighted images, enlargement of the posterior gland, or an isodense homogeneous mass [9]. None of these features were noted in our patient, which led us to exclude the possibility of lymphocytic hypophysitis. With regard to the cause of hypercalcemia, vitamin D overdose or hidden malignancy was excluded because the patient did not show the elevation of either vitamin D3 or PTHrP. 

It was remarkable that there was no evidence of systemic involvement in this case. In Republic of Korea, the first case of neurosarcoidosis without systemic involvement was reported in 1998 by Choi. In contrast to our case, however, steroids did not elicit improvement of symptoms [10]. Though a case of moyamoya-like vasculopathy associated with neurosarcoidosis in Republic of Korea was also reported, it did not accompany any pituitary gland involvement and rather resembled a moyamoya disease according to the MR angiography [11].

In conclusion, this is an interesting case to experience the patient's complaint of intractable headache initially without the abnormal anterior pituitary functions which was presumably diagnosed as neurosarcoidosis due to the characteristic clinical manifestations and the dramatic response to high-dose steroid therapy. Although surgical biopsy of the pituitary lesion was not performed due to the high risk of surgical complications, it should be addressed that patients with both DI and pituitary lesions require thorough examinations with suspecting any inflammatory origin.
